# Identifying deprescribing profiles in older adults from 14 countries using the Patient Deprescribing Typology

**DOI:** 10.1093/geroni/igaf130

**Published:** 2025-11-25

**Authors:** Kristie Rebecca Weir, Vincent D Marshall, Katharina Tabea Jungo, Renata Vidonscky Lüthold, Zsofia Rozsnyai, Sven Streit, Sarah E Vordenberg

**Affiliations:** University of Bern, Institute of Primary Health Care (BIHAM), Bern, Switzerland; University of Sydney, Sydney School of Public Health, Faculty of Medicine and Health, Sydney, New South Wales, Australia; University of Michigan College of Pharmacy, Ann Arbor, Michigan, United States; University of Bern, Institute of Primary Health Care (BIHAM), Bern, Switzerland; Division of Pharmacoepidemiology and Pharmacoeconomics and Center for Healthcare Delivery Sciences, Department of Medicine, Brigham and Women’s Hospital and Harvard Medical School, Boston, Massachusetts, United States; University of Bern, Institute of Primary Health Care (BIHAM), Bern, Switzerland; University of Bern, Institute of Primary Health Care (BIHAM), Bern, Switzerland; University of Bern, Institute of Primary Health Care (BIHAM), Bern, Switzerland; University of Michigan College of Pharmacy, Ann Arbor, Michigan, United States

**Keywords:** Deprescription, Attitudes, Health care communication, Medications, Patient preferences

## Abstract

**Background and Objectives:**

Categorizing older adults by their medication and deprescribing beliefs may support tailored communication and shared decision-making. We aimed to identify latent classes of patients using the Patient Deprescribing Typology.

**Research Design and Methods:**

This cross-sectional study used survey responses from 1,131 primary care patients (≥65 years, ≥5 medications) in 14 countries between May 2022 and December 2023. A latent class analysis was conducted using the 4-item Patient Deprescribing Typology, which captures older adults’ views on medication importance, learning styles, decision-making preferences, and attitudes toward stopping medications. A multinomial logit model was used to compare the latent classes.

**Results:**

Among the 1,131 participants, 55.2% (*n = *624) were female, and on average, participants were taking 7 regular medications (*SD* = 2.3). Three latent classes were identified: (1) *Would consider deprescribing* (*n = *760, 67.2%), (2) *Attached to medications* (*n = *249, 22.0%), and (3) *Defers decision-making to others* (*n = *122, 10.8%). Compared with participants who *Would consider deprescribing*, those *Attached to medications* were significantly less likely to want to deprescribe one of their specific medications (odds ratio [OR] = 0.04, 95% CI: 0.02-0.09, *p* < .001). Participants who *Defer decision-making to others* reported lower trust in their GP (OR = 0.47, 95% CI: 0.32-0.67).

**Discussion and Implications:**

While most participants expressed openness to deprescribing, their preferences for information sources, perceived medication importance, and decision-making roles varied. Our findings highlight the need to account for patient differences in deprescribing attitudes. Typologies offer a practical basis for personalized communication and patient-centered interventions.

Innovation and Translational Significance This study applied a latent class analysis to a 14-country dataset, making it the first study to test the Patient Deprescribing Typology across multiple countries with patients reflecting on their actual medications. We identified 3 profiles based on patients’ attitudes toward medications, information-seeking, and deprescribing preferences: *Would consider deprescribing* (most common), *Attached to medications*, and *Defers decision-making to others*. Identifying these typologies provides a practical foundation for tailoring patient-clinician communication and designing patient-centered deprescribing interventions across diverse healthcare systems.

## Background and objectives

The increasing use of medications among older adults has heightened global concern about polypharmacy and its associated risks.^1^ Polypharmacy, usually defined as the use of 5 or more regular medications, is common among older adults and is associated with adverse effects, hospitalizations, and treatment burden.^2^^,^^3^ International efforts to promote safe medication use have emphasized deprescribing – reducing or stopping medications that may no longer be necessary or beneficial – as a key strategy to minimize medication-related harm.^4^^,^^5^ However, deprescribing remains inconsistently adopted in practice.^6^ Older adults may be resistant to deprescribing due to beliefs in the necessity or effectiveness of their medications, fear that stopping a medication could worsen their health, or trust in their doctor’s original decision to prescribe.^6–9^

Recent studies suggest substantial variation in how older adults perceive and respond to recommendations of deprescribing, with acceptance rates varying widely across countries and settings.^10–12^ For example, in the larger study underpinning this analysis, patients’ interest in deprescribing at least one of their specific medications ranged from 23% in Bulgaria to 75% in Italy, indicating that different contexts may shape preferences for deprescribing.^13^ This variability highlights the need to understand how sociocultural, healthcare system, and individual-level factors influence medication-related decision-making. Cross-national, quantitative research can systematically capture patterns in patients’ attitudes toward medications and preferences for deprescribing to identify key subgroups with distinct decision-making profiles.

Typologies have been used to identify patterns in how older adults perceive and manage their medications. Previous typologies have focused on deprescribing cardiovascular or cardiometabolic medications,^14^^,^^15^ self-management of medications,^16^ and decision-making preferences.^17^^,^^18^ In the context of deprescribing, the Patient Deprescribing Typology offers a structured way to consider heterogeneity, moving beyond the notion that patients are either uniformly for or against deprescribing, or that each patient is entirely unique. Examining the typology across diverse countries shows whether these profiles are universal or context-specific, informing how to tailor communication strategies and interventions across different healthcare systems and cultural settings.

While previous work applied the Patient Deprescribing Typology to hypothetical vignette-based scenarios in 4 countries,^19^^,^^20^ its applicability when older adults consider their actual medications across diverse international populations remains unexamined. To address this gap, we applied latent class analysis to a 14-country dataset^13^ to examine the cross-national relevance of the typology and to identify and describe meaningful subgroups of patients based on their attitudes toward medications, information-seeking behavior, and deprescribing preferences.

## Research design and methods

### Study design and participants

General practitioners (GPs) recruited through the European General Practice Research Network were national coordinators, leading recruitment in their respective countries with a recruitment target of 100 older adults per country. National coordinators enrolled additional GPs, who consecutively invited eligible patients to participate. Recruitment occurred across 17 sites in 14 countries between May 2022 and December 2023. This study was part of a larger survey^13^ which aimed to understand older adults’ attitudes toward deprescribing, with further details available elsewhere.^13^^,^^21^ Older adults were eligible for the survey if they were aged 65 years or older and taking 5 or more regular medications. Individuals were excluded if they were unable to provide informed consent or were not residing in one of the participating countries.

The study followed the principles of the Declaration of Helsinki^22^ and received ethical approval from the ethics committee of Bern, Switzerland (*Kantonale Ethikkommission Bern*) (Project-ID 2022–00035). National coordinators obtained local ethics approval where necessary. To comply with European privacy regulations, the study met the requirements of the General Data Protection Regulation through anonymization and data source protection. Informed consent was obtained electronically; participants indicated their consent by selecting “yes” to a consent question before proceeding with the survey. Those who selected “no” were unable to access the survey.

### Questionnaire development

The Patient Deprescribing Typology was developed through a program of research that included a qualitative study in Australia involving interviews with 30 older adults and 15 caregivers^7^ and 2 subsequent quantitative studies.^19^^,^^20^ These quantitative studies used survey data from older adults recruited online in Australia, the Netherlands, the United Kingdom, and the United States and one of the studies^20^ applied latent class analysis to identify distinct groups based on patients’ beliefs and preferences related to deprescribing. The typology captures 4 key domains: beliefs about medication importance, learning style, decision-making preferences, and attitudes toward deprescribing (Table 1). Participants responded to 4 questions, each corresponding to one of these domains.

**Table 1.** Domains, statements, and keywords of the Patient Deprescribing Typology.

Domain	Statement^a^	Keywords
**Beliefs about importance**	What do you think about the medications you take?	
	My medications are important, they keep me alive and help me live well.	Important
	My medications do what they are supposed to do.	Effective
	I don’t really care much about my medications, I take them as my doctor tells me to.	Not important
**Learning style**	How do you get information about your medications?	
	My doctor and I talk about my medications together.	Discuss with doctor
	I know about my medications—I ask my doctor or read the information leaflet or search online.	Multiple sources
	I don’t know much about my medications.	Not informed
**Decision-making preferences**	How do you make decisions about your medications?	
	*I want to be informed, but* I trust my doctor to make decisions about my medications.	Decision by doctor
	I make decisions about the medications I take, or share the decision with my doctor.	Decision by self
	Other people (eg, my doctor *or my partner*) make decisions for me about my medications.	Decision by other
**Attitudes toward deprescribing**	What do you think about the idea of stopping *or reducing the dose of* one or more of your medications?	
	If my doctor said that it is possible to stop *or reduce the dose of* a medication that would be ok with me.	Open to deprescribing
	I wish I did not take so many medications and I would stop *or reduce the dose of* my medications if I could.	Prefer deprescribing
	I would not *like* to stop any of my medications *or reduce the dose.*	Continue medications

Text in italics denotes changes from the previous wording.^20^

a Participants were asked: “For each of the following, please select the statement that best aligns with your views.”

In this study, minor wording adjustments were made to the Patient Deprescribing Typology by a multidisciplinary research team with expertise in general practice, pharmacy, health communication, and public health to improve clarity and to explicitly include “dose reduction” within the statements related to deprescribing. The Patient Deprescribing Typology questions were translated into more than 10 languages, using a forward- and back-translation process by the respective national coordinators in the participating countries. Patients completed the questionnaire either online via the REDCap survey platform or on paper.

We assessed patients’ interest in deprescribing using the question: “*Thinking about your current medication list, are there any medications that you would like to stop taking or reduce the dose of?*” We collected demographic information (gender, education, health literacy,^23^^,^^24^ financial status, self-rated health^25^) and the number of medications. We also assessed patients’ trust in their GP using the abbreviated Wake Forest Trust in Physician Scale,^26^ satisfaction with medications^27^ and attitudes toward deprescribing.^27^

### Statistical analysis

Our data were summarized with the mean and standard deviation for continuous variables and counts and percentages for categorical data. Means were chosen over medians in some cases, as the medians did not reveal sufficient information to compare between groups, given that our numeric data was primarily from Likert scale responses. Numeric data were compared with independent samples *t*-tests, and categorical data were compared with Fisher’s exact tests.

We used a statistical method called latent class analysis^28^ to see if we could group older adults into different types based on how they responded to 4 questions about their attitudes toward medications and deprescribing. This method helps identify patterns in people’s responses by identifying subgroups or “classes” of individuals who tend to answer in similar ways. These subgroups can then be used to explore whether people with similar attitudes also share other characteristics, such as their health status, medication use, or willingness to stop a medication.

Our latent classes were first observed by using Bayesian Information Criterion (BIC) values to determine optimal numbers of underlying classes. We used replications of 100 models to ensure that our BIC minimization was global, rather than local. In the process of estimating the latent classes, we used many variables in a multinomial model, predicting categorical variables describing the attitudes toward medications and deprescribing by education, gender, attitudes toward deprescribing medications, average trust in general practitioners, and self-reported health. We excluded participants who reported missing data for variables included in the latent class analysis model as these participants were unable to be categorized. A multinomial regression was used both in estimating the latent classes and in a table presenting odds ratios and 95% confidence intervals comparing the classes. A Type I error was set as 0.05 as all comparisons were individual. Statistical analyses were performed with R statistical software (v. 4.2.2).^29^ The poLCA package in R was used in the estimation of latent classes.^30^

## Results

### Characteristics of participants

There were 1,423 older patients who started answering the questionnaire, of whom 1,340 provided consent and completed more than 5 questions (including 4 eligibility questions: informed consent, aged ≥65 years, taking ≥5 medications regularly, and residing in one of the participating countries). A total of 209 participants were excluded due to missing data, resulting in a final analytical sample of 1,131 participants.

Participants (*n = *1,131) were frequently female (*n = *624, 55.2%), reported secondary school as the highest education level (*n = *478, 42.3%), and were “extremely” or “quite a bit” confident in filling out medical forms (*n = *607, 53.7%) (Table 2). Participants were taking a mean of 7 (*SD* = 2.3) regular medications. Many (*n = *514, 45.4%) reported average health, and approximately one-half (*n = *534, 49.3%) reported establishing a relationship with their GP within the past 10 years.

**Table 2.** Participant characteristics overall and by latent class (*N = *1,131).^a^

Participant characteristics	Number of participants (%)				*p*-value
	Overall	Class 1 “Would consider deprescribing”	Class 2 “Attached to medications”	Class 3 “Defers to others”	
	(*N = *1131)	(*n = *760)	(*n = *249)	(*n = *122)	
**Gender, *n* (%)**					.03
** Male**	507 (44.8)	347 (45.7)	96 (38.6)	64 (52.5)	
** Female**	624 (55.2)	413 (54.3)	153 (61.4)	58 (47.5)	
**Education, *n* (%)**					<.001
** Primary or none**	316 (27.9)	165 (21.7)	75 (30.1)	76 (62.3)	
** High school or vocation training**	478 (42.3)	324 (42.6)	113 (45.4)	41 (33.6)	
** University**	337 (29.8)	271 (35.7)	61 (24.5)	5 (4.1)	
**Geography, *n* (%)**					<.001
** Urban**	605 (53.6)	404 (53.2)	137 (55.2)	64 (52.5)	
** Suburban**	312 (27.6)	216 (28.5)	78 (31.5)	18 (14.8)	
** Rural**	212 (18.8)	139 (18.3)	33 (13.3)	40 (32.8)	
**Live alone, *n* (%)**	422 (37.4)	270 (35.6)	94 (37.9)	58 (47.5)	.043
**Housing, *n* (%)**					.099
** Own**	885 (78.6)	594 (78.5)	187 (75.7)	104 (85.2)	
** Rent**	241 (21.4)	163 (21.5)	60 (24.3)	18 (14.8)	
**Financial, *n* (%)**					<.001
** Great difficulty**	75 (6.7)	28 (3.7)	22 (8.8)	25 (21.0)	
** Some difficulty**	404 (35.9)	254 (33.6)	85 (34.1)	65 (54.6)	
** Quite easily**	381 (33.9)	270 (35.7)	95 (38.2)	16 (13.4)	
** No problems**	264 (23.5)	204 (27.0)	47 (18.9)	13 (10.9)	
**Country, *n* (%)**					<.001
** Belgium**	99 (8.8)	74 (9.7)	21 (8.4)	4 (3.3)	
** Bulgaria**	89 (7.9)	51 (6.7)	35 (14.1)	3 (2.5)	
** Croatia**	25 (2.2)	15 (2.0)	6 (2.4)	4 (3.3)	
** Germany**	72 (6.4)	47 (6.2)	19 (7.6)	6 (4.9)	
** Hungary**	94 (8.3)	70 (9.2)	19 (7.6)	5 (4.1)	
** Ireland**	24 (2.1)	16 (2.1)	8 (3.2)	0 (0.0)	
** Israel**	57 (5.0)	47 (6.2)	10 (4.0)	0 (0.0)	
** Italy**	85 (7.5)	44 (5.8)	16 (6.4)	25 (20.5)	
** Netherlands**	179 (15.8)	136 (17.9)	32 (12.9)	11 (9.0)	
** Poland**	104 (9.2)	51 (6.7)	7 (2.8)	46 (37.7)	
** Portugal**	85 (7.5)	50 (6.6)	29 (11.6)	6 (4.9)	
** Spain**	86 (7.6)	63 (8.3)	17 (6.8)	6 (4.9)	
** Sweden**	99 (8.8)	73 (9.6)	23 (9.2)	3 (2.5)	
** Switzerland**	33 (2.9)	23 (3.0)	7 (2.8)	3 (2.5)	
**Relationship with general practitioner (GP)**					.410
**Has an established general practitioner, *n* (%)**	1,088 (97.4)	733 (97.9)	239 (96.4)	116 (96.7)	
**Duration of relationship with GP, *n* (%)**					.001
** Less than 10 years**	534 (49.3)	369 (50.5)	108 (45.2)	57 (49.6)	
** 10-19 years**	318 (29.3)	205 (28.1)	75 (31.4)	38 (33.0)	
** 20-29 years**	141 (13.0)	108 (14.8)	28 (11.7)	5 (4.3)	
** 30 or more years**	91 (8.4)	48 (6.6)	28 (11.7)	15 (13.0)	
**Trust in GP, mean (*SD*)**	3.8 (0.6)	3.8 (0.6)	4.0 (0.6)	3.6 (0.7)	
***Health-related characteristics***					
**Health status, *n* (%)**					<.001
** Excellent, very good, or good**	439 (38.8)	302 (39.7)	124 (49.8)	13 (10.7)	
** Average**	514 (45.4)	351 (46.2)	103 (41.4)	60 (49.2)	
** Poor**	178 (15.7)	107 (14.1)	22 (8.8)	49 (40.2)	
**Confidence filling out medical forms, *n* (%)**					<.001
** Extremely**	227 (20.1)	180 (23.7)	42 (17.1)	5 (4.1)	
** Quite a bit**	380 (33.7)	273 (35.9)	90 (36.6)	17 (13.9)	
** Somewhat**	280 (24.8)	185 (24.3)	66 (26.8)	29 (23.8)	
** A little bit**	150 (13.3)	79 (10.4)	27 (11.0)	44 (36.1)	
** Not at all**	91 (8.1)	43 (5.7)	21 (8.5)	27 (22.1)	
***Medication-related characteristics***					
**Number of regular medications, mean (*SD*)**	7 (2.3)	7 (2.4)	7 (2.4)	7 (2.1)	.021
**Medication management, *n* (%)**					<.001
** Independent**	984 (87.4)	698 (92.2)	215 (87.0)	71 (58.2)	
** Support needed**	142 (12.6)	59 (7.8)	32 (13.0)	51 (41.8)	
**Satisfied with current medications, *n* (%)**					<.001
** Strongly agree**	272 (24.1)	164 (21.7)	105 (42.3)	3 (2.5)	
** Agree**	642 (57.0)	454 (60.0)	120 (48.4)	68 (55.7)	
** Don’t know**	171 (15.2)	110 (14.5)	19 (7.7)	42 (34.4)	
** Disagree**	40 (3.5)	29 (3.8)	3 (1.2)	8 (6.6)	
** Strongly disagree**	2 (0.2)	0 (0)	1 (0.4)	1 (0.8)	
**Would like to stop medication and see how I feel without it, *n* (%)**					<.001
** Strongly agree**	163 (14.4)	126 (16.6)	13 (5.2)	24 (19.7)	
** Agree**	380 (33.6)	305 (40.1)	36 (14.5)	39 (32.0)	
** Don’t know**	300 (26.5)	179 (23.6)	83 (33.3)	38 (31.1)	
** Disagree**	221 (26.5)	120 (15.8)	84 (33.7)	17 (13.9)	
** Strongly disagree**	67 (5.9)	30 (3.9)	33 (13.3)	4 (3.3)	
**Would like to deprescribe specific medication, *n* (%)**					<.001
** Yes**	514 (45.4)	413 (54.3)	10 (4.0)	91 (74.6)	
** No**	617 (54.6)	347 (45.7)	239 (96.0)	31 (25.4)	
**Comfortable talking with GP about medication changes, *n* (%)**					<.001
** Strongly agree**	486 (43.6)	347 (46.3)	123 (50.8)	16 (13.1)	
** Agree**	490 (44.0)	350 (46.7)	85 (35.1)	55 (45.1)	
** Don’t know**	102 (9.2)	44 (5.9)	19 (7.9)	39 (32.0)	
** Disagree**	30 (2.7)	6 (0.8)	12 (5.0)	12 (9.8)	
** Strongly disagree**	6 (0.5)	3 (0.4)	3 (1.2)	0 (0)	
***Patient Deprescribing Typology***					
**Beliefs about importance, *n* (%)**					<.001
** Important**	509 (45.0)	279 (36.7)	193 (77.5)	37 (30.3)	
** Effective**	472 (41.7)	388 (51.1)	50 (20.1)	34 (27.9)	
** Not important**	150 (13.3)	93 (12.2)	6 (2.4)	51 (41.8)	
**Learning style, *n* (%)**					<.001
** Discuss with doctor**	589 (52.1)	411 (54.1)	173 (69.5)	5 (4.1)	
** Multiple sources**	406 (35.9)	339 (44.6)	64 (25.7)	3 (2.5)	
** Not informed**	136 (12.0)	10 (1.3)	12 (4.8)	114 (93.4)	
**Decision-making preferences, *n* (%)**					<.001
** Decision by doctor**	855 (75.6)	612 (80.5)	211 (84.7)	32 (26.2)	
** Decision by self**	155 (13.7)	118 (15.5)	34 (13.7)	3 (2.5)	
** Decision by other**	121 (10.7)	30 (3.9)	4 (1.6)	87 (71.3)	
**Attitudes toward deprescribing, *n* (%)**					<.001
** Open to deprescribing**	579 (51.2)	442 (58.2)	66 (26.5)	71 (58.2)	
** Prefer deprescribing**	364 (32.2)	318 (41.8)	0 (0)	46 (37.7)	
** Continue medications**	188 (16.6)	0 (0)	183 (73.5)	5 (4.1)	

a The missing data were less than or equal to 4% for all variables.

When asked about their beliefs regarding medications, participants frequently highlighted the importance of their medications (*n = *509, 45.0%) or referred to their effectiveness (*n = *472, 41.7%). Over half (*n = *589, 52.1%) reported discussing their medications with their doctor to learn more, and most participants (*n = *855, 75.6%) preferred to be informed but trusted their doctor to make medication decisions. Participants were generally open to deprescribing, with one-half (*n = *579, 51.2%) expressing openness to the idea and one-third (*n = *364, 32.2%) preferring to deprescribe if possible.

### Characteristics of latent classes

We identified 3 latent classes in relation to the Patient Deprescribing Typology. Most participants (*n* = 760, 67.2%) were open to stopping their medications (Class 1: *Would consider deprescribing*), whereas 22.0% (*n* = 249) were attached to and highly valued their medications (Class 2: *Attached to medications*), and 10.8% (*n* = 122) preferred to defer decisions to others (Class 3: *Defers decision-making to others*).

*Would consider deprescribing* (Class 1) participants were most often female (*n = *413, 54.3%), and many reported earning a high school diploma (*n = *324, 42.6%). Participants thought that their medications were effective (*n = *388, 51.1%), they discussed their medications with their doctor (*n = *411, 54.1%) or used multiple sources of information to learn about medications (*n = *339, 44.6%). They trusted their doctor to make decisions about their medications (*n = *612, 80.5%), and they were open to (*n = *442, 58.2%) or preferred (*n = *318, 41.8%) deprescribing.

Class 2: *Attached to medications* participants were most often female (*n = *153, 61.4%). They most frequently reported obtaining a high school education or vocational training (*n = *113, 45.4%). Participants reported that their medications were highly important (*n = *193, 77.5%), they discussed with their doctor to learn about medications (*n = *173, 69.5%), they trusted their doctor to make decisions about their medicines (*n = *211, 84.7%), and they preferred to continue their medications (*n = *183, 73.5%).

Class 3: *Defers decision-making to others* participants were most often male (*n = *64, 52.5%). The majority (*n = *76, 62.3%) had completed a primary school education. Participants considered that their medications were not important (*n = *51, 41.8%) and most reported not to know much about their medications (*n = *114, 93.4%). They tended to defer medication-related decisions to others (*n = *87, 71.3%) and were generally open to deprescribing (*n = *71, 58.2%).

### Comparison across latent classes

We compared each latent class to Class 1: *Would consider deprescribing* as it included the largest number of participants (Table 3). Compared with participants in Class 1: *Would consider deprescribing*, participants in Class 2: *Attached to medications* were more likely to attend primary school (as opposed to university) (odds ratio [OR] = 2.02, 95% CI: 1.09, 3.75) and were less likely to share a preference to deprescribe a specific medication they were currently taking (OR = 0.04, 95% CI: 0.02, 0.09). Participants in Class 3: *Defers decision-making to others* were less likely to be female (OR = 0.54, 95% CI: 0.33, 0.90) and reported less trust in their GP (OR = 0.47, 95% CI: 0.32, 0.67). They were more likely to report primary school (OR = 16.44, 95% CI: 5.49, 49.19) or high school education (OR = 6.86, 95% CI: 2.26, 20.76) and average (OR = 3.02, 95% CI: 1.41, 6.43) or poor (OR = 7.61, 95% CI: 3.26, 17.77) as opposed to excellent, very good, or good health status (Table 3).

**Table 3.** Multinomial logit model comparing deprescribing latent classes.

Variable	Class 2 *“Attached to medications”* vs Class 1 *“Would consider deprescribing”*		Class 3 *“Defers to others”* vs Class 1 *“Would consider deprescribing”*	
	OR (95% CI)	*p*	OR (95% CI)	*p*
**Demographic characteristics**				
**Gender**				
** Male**	REF	REF	REF	REF
** Female**	1.40 (0.90, 2.19)	.139	0.54 (0.33, 0.90)	.018
**Education**				
** Primary school or none**	2.02 (1.09, 3.75)	.025	16.44 (5.49, 49.19)	<.001
** High school or vocational training**	1.35 (0.82, 2.21)	.238	6.86 (2.26, 20.76)	.001
** University**	REF	REF	REF	REF
**Relationship with general practitioner (GP)**				
**Trust in GP**	1.19 (0.82, 1.74)	.364	0.47 (0.32, 0.67)	<.001
***Health-related characteristics***				
**Health status**				
** Excellent, very good, or good**	REF	REF	REF	REF
** Average**	0.75 (0.47, 1.19)	.225	3.02 (1.41, 6.43)	.004
** Poor**	0.53 (0.25, 1.15)	.110	7.61 (3.26, 17.77)	<.001
**Would like to deprescribe specific medication**				
** Yes**	0.04 (0.02, 0.09)	<.001	1.88 (0.98, 3.58)	.056
** No**	REF	REF	REF	REF

## Discussion and implications

The 3 latent classes identified in this study closely align with the original Patient Deprescribing Typology “Qualitative interviews” study, and observed in the “Quantitative development” study, in which participants were asked to select from one of 3 pre-defined Patient Deprescribing Typologies (Table 4). In alignment with the “Quantitative development” study,^19^ we found that the most common typology was *Would consider deprescribing* followed by *Attached to medications*, then *Defers decision-making to others*. An important finding given that this was the first time the Patient Deprescribing Typology was studied across a larger number of countries.

**Table 4.** Development of the Patient Deprescribing Typology.

Title summary	“Qualitative interviews”	“Quantitative development”	“4-country vignette LCA”	“14-country LCA”
**Citation**	Weir et al., 2018^7^	Weir et al., 2024^28^	Weir et al., 2025^20^	Current study
**Objective**	To explore the reasons behind variation in older adults’ attitudes and preferences related to medication deprescribing	To develop a quantitative Patient Deprescribing Typology measure that could be incorporated into research	To define distinct latent classes of older adults via the Patient Deprescribing Typology	To identify distinct latent classes of older adults via the Patient Deprescribing Typology
**Study population**	Adults 75 years and older (*n = *30) and companions (*n = *15) in Australia recruited from primary care and hospital settings	Adults 65 years and older (*n = *4,688) in Australia, the Netherlands, the United Kingdom, and the United States recruited online	Adults 65 years and older (*n = *2,250) in Australia, the Netherlands, the United Kingdom, and the United States recruited online	Adults 65 years and older taking 5 or more medications (*n = *1,131) in 14 counties recruited from primary care
**Methodology**	In-depth semi-structured interviews followed by a framework analysis, guided by a theoretical shared decision-making framework (Jansen et al., 2016)	Participants selected one of 3 pre-defined Patient Deprescribing Typologies. Descriptive statistics and multinomial logistic regression analyses were conducted.	Participants selected one choice for each of the 4 individual Patient Deprescribing Typology questions. A latent class analysis was conducted.	Participants selected one choice for each of the 4 individual Patient Deprescribing Typology questions. A latent class analysis was conducted.
**Results**	Three typologies were identified: *Would consider deprescribing* *Attached to medications* *Defers to others*	Participants selected the following typologies: *Would consider deprescribing* (*n = *2464, 53%) *Attached to medications* (*n = *1446, 30%) *Defers medication decision-making to others* (*n = *778, 17%)	Four latent classes were identified: *Trusts their doctor* (*n = *935, 41.6%) *Makes own decisions* (*n = *680, 30.2%) *Avoids deprescribing* (*n = *349, 15.5%) *Medications not important* (*n = *286, 12.7%)	Three latent classes were identified: *Would consider deprescribing* (*n = *760, 67.2%) *Attached to medications* (*n = *249, 22.0%) *Defers decision-making to others* (*n = *122, 10.8%)

*Source:* Adapted from Weir et al. (2025).^20^

We previously reported the results of a “4-country vignette latent class analysis (LCA)” in which we used the same methodology as this study (ie, asking participants to select one choice for each of the 4 individual Patient Deprescribing Typology questions) among 2,250 participants from Australia, the Netherlands, the United Kingdom, and the United States. While 3 of the classes were similar to those identified here, that study also described a fourth latent class, *Makes own decisions*, characterized by high autonomy, independent decision-making, and a preference for managing medications themselves. Individuals in this class were more often from the Netherlands, reported lower trust in their doctors, and expressed a stronger preference for deprescribing. There were several key differences between that study and the current one. The previous study reported higher overall agreement with deprescribing, likely because participants were asked to respond to a hypothetical vignette rather than reflect on their own medications. Recruitment in the previous study also relied on an online panel, which attracted participants who were generally healthier and more accustomed to making their own decisions about medications. In contrast, participants in the current study were recruited through GPs in primary care and were required to be taking at least 5 regular medications. This approach yielded a sample of older adults with more varied health status, higher medication use, a stronger perceived need for their medications, and most likely greater trust in their doctors. The qualitative interview study^7^ similarly recruited participants through general practices and a hospital rehabilitation ward to capture older adults with varying health and more complex medication use, which may explain why the current findings align more closely with the original qualitative typology.

The multinomial regression found that certain demographic and clinical characteristics were more common in each class: for example, lower education level obtained and reduced preference to deprescribe in the *Attached to medications* class, and male gender, poorer health, lower trust in the GP, and lower education level obtained in the *Defers decision-making to others* class. Rather than serving to predict or classify patients, these characteristics offer insights into different patient typologies, supporting clinicians in understanding patients’ perspectives and adjusting their communication as needed. For example, with patients who are *Attached to medications*, GPs could focus on discussing a clear timeframe for medication use, limiting deprescribing conversations to high-risk situations, addressing emotions, fears, and concerns, and emphasizing the safety and potential benefits of stopping a medication. For patients who *Would consider deprescribing*, GPs might highlight that not starting a medication may be an option, openly acknowledge and discuss uncertainty, and routinely review medications and revisit earlier decisions as circumstances change. For patients who *Defer decision-making to others*, it may be helpful to first identify who the patient wants to involve in decision-making (eg, a family member or caregiver), use open-ended questions and empathic responses to explore their views, and look for appropriate opportunities to introduce deprescribing. These strategies provide examples of how typologies can be used in practice to guide tailored conversations that support shared decision-making in routine primary care.

Research is underway to assess the typology’s predictive utility. A cluster-randomized controlled trial in Swiss primary care focused on deprescribing inappropriate proton pump inhibitors,^31^^,^^32^ is evaluating whether the typology can identify patient subgroups more or less likely to engage in deprescribing, thereby assessing its ability to predict actual deprescribing uptake. In parallel, ongoing work is occurring to develop a 2-question version for use in clinical practice that identifies who the patient wants to involve in deprescribing decisions and their preferences toward deprescribing their current medications. Additional work is exploring how older adults respond to the typology when considering a specific medication versus their entire medication regimen. Longitudinal research is also needed to understand how typologies relate to deprescribing behavior over time and whether these preferences are stable or modifiable. Together, these findings could inform more effective, patient-centered deprescribing strategies across diverse clinical settings.

Although formal cross-national comparisons were beyond the scope of the current analysis, it is plausible that differences in healthcare systems, trust in healthcare professionals, attitudes toward medication-related decision making, and national policy approaches to medication management shape attitudes toward deprescribing. Future research may explore these cross‑national differences in greater depth to better tailor deprescribing strategies.

### Limitations

This study has several limitations. Participants’ stated preferences about deprescribing may not reflect actual behavior in clinical settings. Because data were collected anonymously across multiple countries, response rates could not be tracked. Although GPs were asked to recruit patients consecutively, selection bias is possible, particularly if GPs chose patients who were more receptive to medication changes or with whom they had stronger relationships. This may have inflated levels of reported trust and willingness to deprescribe. Additionally, we did not collect exact patient ages, only whether they were 65 or older, which limited our ability to explore age-related differences in attitudes.

Latent class analysis is a valuable tool for identifying unobserved patterns in the data; however, it relies on model fit statistics and researcher judgment to determine the number and interpretation of classes. As a probabilistic approach, it does not guarantee accurate classification at the individual level. Finally, we were unable to determine whether participants in different countries varied in their distribution across the 3 latent classes, as including all 14 countries was not feasible within the latent class analysis modeling approach.

## Conclusion

Through latent class analysis of older adults across 14 countries, we identified 3 distinct Patient Deprescribing Typology classes, reflecting the varied attitudes and preferences older adults hold toward their medications. These empirically derived findings from primary care patients show the importance of recognizing patient heterogeneity in deprescribing decisions. Identifying these typologies offers a practical foundation for tailoring communication and developing more patient-centered deprescribing interventions that can be adapted across diverse international healthcare settings.

## Data Availability

The data for this study are available to other researchers on request. The data will be made available for scientific research purposes after the proposed analysis plan has been approved by the core study team. Data and documentation will be made available through a secure file exchange platform after approval of the proposal. In addition, a data transfer agreement must be signed (which defines obligations that the data requester must adhere to regarding privacy and data handling). For data access, please contact the corresponding author.
